# The Production of γ-Aminobutyric Acid from Free and Immobilized Cells of *Levilactobacillus brevis* Cultivated in Anaerobic and Aerobic Conditions

**DOI:** 10.3390/microorganisms10112184

**Published:** 2022-11-03

**Authors:** Teresa Zotta, Immacolata Faraone, Marilisa Giavalisco, Eugenio Parente, Ludovica Lela, Livia Vanessa Storti, Annamaria Ricciardi

**Affiliations:** 1 Scuola di Scienze Agrarie, Alimentari, Forestali ed Ambientali (SAFE), Università degli Studi della Basilicata, 85100 Potenza, Italy; 2 Dipartimento di Scienze (DIS), Università degli Studi della Basilicata, 85100 Potenza, Italy; 3 Spinoff BioActiPlant s.r.l., Viale Dell’ateneo Lucano 10, 85100 Potenza, Italy

**Keywords:** γ-aminobutyric acid, *Levilactobacillus brevis*, aerobiosis, immobilized cells, recycling, starvation

## Abstract

γ-aminobutyric acid (GABA) has several beneficial effects on human health. GABA may be produced via chemical synthesis or through microbial metabolism, and *Levilactobacillus brevis* is recognized as a GABA-producing species. In this study, 11 *Lvb. brevis* strains were screened for GABA production, and the best producers were selected to verify the effect of aerobic (AE) and respiratory (RS) cultivations on growth parameters, biomass, and GABA accumulation. *Lvb. brevis* LB12 was then used to evaluate the combined effect of the incubation atmosphere (anaerobiosis vs. aerobiosis), cell protection (free vs. immobilized cells), and cell recycling (fresh vs. starved cells) on GABA production. Glutamate (GLU) consumption and GABA accumulation were detected by Thin-layer Chromatography (TLC) and RP-HPLC analyses. The ability to produce GABA was widespread among the strains. AE and RS growth improved biomass production, but oxygen availability impaired GLU to GABA conversion, and the anaerobically growing cells had the highest GABA productivity. Immobilized strains had lower efficiency in both GLU uptake and conversion compared to free cells, probably due to the poor diffusion in alginate beads. The use of resting cells allowed further GABA production without the cultivation step, but cell activity was exhausted after three cycles of reutilization. *Lvb. brevis* LB12 is an excellent GABA producer, and AE cultivation can be exploited to improve the final cell density; however, the conditions for boosting GLU to GABA conversion and cell regeneration need to be further investigated.

## 1. Introduction

γ-aminobutyric acid (GABA) is a non-proteinogenic amino acid recognized as the key inhibitory neurotransmitter in mammalian nervous systems. GABA has several physiological functions (e.g., the promotion of brain development, the regulation of neurological disorders, and hypotensive, analgesic, antianxiety, antidiabetic, and diuretic effects), and its consumption, both as a pharmaceutical or dietary supplement, may result in different benefits to human health [1,2,3]. GABA occurs in several cereal and vegetable products (including some fermented foods, e.g., kimchi, tempeh, tea [4,5,6]), but its content is variable and often not high enough to reach beneficial effects, and different strategies that aim to improve its concentration in foods are gaining interest.

GABA may be produced via chemical synthesis or through the irreversible decarboxylation of glutamate by pyridoxal 5′-phosphate (PLP)-dependent microbial glutamate decarboxylases [1,2,7]. Bioconversion is expected to overcome the chemical processes (data of the GABA global market, forecast period 2022–2029; [8]) because it provides a natural and sustainable product that can be used as a supplement in functional foods, drugs, and cosmetics.

Lactic acid bacteria (LAB) are recognized as potential GABA producers and, currently, several strains belonging to the species *Levilactobacillus brevis*, *Lvb. buchneri*, *Lacticaseibacillus paracasei*, *Lactiplantibacillus plantarum*, *Lactobacillus delbrueckii* subsp. *bulgaricus*, *Lb. helveticus,* and *Lactococcus lactis* have been investigated and characterized for their capability to produce GABA in synthetic media and/or food matrices [1,7,9].

In LAB, the glutamate decarboxylase (GAD) and glutamate/GABA antiporter system (GadC) (both encoded by the *gad* operon) promote the respective decarboxylation of glutamate to GABA (with the consumption of a proton H^+^ and the production of CO_2_) and the external secretion of GABA. Most LAB harbor only the GAD-encoding genes, while just a few strains (mainly belonging to *Lvb. brevis* and *Lvb. buchneri*) have the complete *gad* operon [7,10]. In contrast with other GABA-producers (i.e., *Escherichia coli*), LAB do not possess the GABA shunt system [7]. Besides genetic equipment, other factors may affect the functionality of GABA pathways [7]. An efficient bioconversion requires a high cell density and, therefore, the optimal growth conditions of GABA-producers (e.g., temperature, substrate composition, carbon source) should be satisfied. Moreover, the parameters affecting the catalytic activity of GAD (e.g., pH values, PLP cofactor, glutamate content) should also be monitored [2,7]. On the other hand, the downstream processes needed for the separation and purification of GABA from culture broth are often expensive and may impair the large-scale production and the marketability of GABA and/or GABA-supplemented foods. Therefore, strategies to boost bioconversion efficiency and GABA accumulation are needed to reduce the production costs and promote industrial applications.

To our knowledge, the effect of oxygen and energy metabolism on GABA production has been poorly investigated in LAB [11]. It is known that in some strains (see Pedersen et al. [12] and Zotta et al. [13] for reviews), the shift from anaerobic fermentative growth to aerobic and/or respirative metabolism resulted in increased biomass and cell robustness. Aerobic cultivation, then, may be a natural strategy to enhance the final cell density and improve GABA biocatalysis in LAB.

Moreover, the use of resting cells (non-growing but metabolically active) may also provide further advantages over the use of growing cells (avoiding the limitations of the fermentation process) and/or purified enzymes (avoiding the constraints of separation techniques), as they may be recycled and reused for the bioconversion process [14,15]. To date, the use of resting cells has been exploited to improve GABA production from *Lvb. brevis* strains in buffered systems [14,15,16], but suitable regeneration protocols are needed to maintain the catalytic activity over extended periods.

In this study, 11 strains of *Lvb. brevis* were screened for their capability to produce GABA in glutamate-supplemented medium. Five selected strains were cultivated under anaerobic, aerobic, and respiratory conditions to evaluate the effect of cultivation on the biomass yield and GABA production. The best GABA-producer, *Lvb. brevis* LB12, was further used to evaluate the combined effect of the atmosphere of incubation (anaerobiosis vs. aerobiosis), cell protection (free vs. immobilized cells), and cell recycling (fresh vs. starved cells) on GABA production. GABA was qualitatively detected by Thin-layer Chromatography (TLC) and quantified by High-performance Liquid Chromatography (HPLC), and a correlation among the two techniques was also conducted.

## 2. Materials and Methods

### 2.1. Strains and Culture Conditions

*Levilactobacillus brevis* PA11S, PB13L, A7, A4, B02, B17, B29, F02, B24, and B25 (from sourdough) and TO62 (raw milk) were used in this study. The strains were maintained as freeze-dried stocks (11% *w*/*v* skim milk with 0.1% *w*/*v* ascorbic acid) in the Culture Collection of Industrial Microbiology Laboratory, Università degli Studi della Basilicata, and propagated in MRS broth, pH 6.8 (MRS; Oxoid, Thermo Fisher Scientific Inc., Basingstoke, Hampshire, United Kingdom) or modified WMB (mWMB; the composition is tryptone, 5 g/L; KH_2_PO_4_, 1 g/L; yeast extract, 10 g/L; glucose, 10 g/L; MgSO_4_∙7H_2_O, 0.2 g/L, MnSO_4_∙H_2_O, 0.05 g/L, and Tween 80 0.5 mL; [17]), according to the experimental trials. 

### 2.2. Screening for Glutamate Decarboxylase Activity and GABA Production

Cultures grown in MRS (cultivated for 16 h at 30 °C) were standardized (final absorbance at 650 nm, A_650_, of 3.0; SmartSpec™ Plus Spectrophotometer, Bio-Rad Laboratories Inc., Milan, Italy) and used to inoculate (1% *v*/*v*) MRS with or without monosodium glutamate (10 g/L; MSG) supplementation (hereinafter MRS-G and MRS, respectively). After 24 h and 48 h of incubation (30 °C, static condition), the pH (CyberScan pH11/110, Oakton Instruments, Vernon Hills, IL, USA; Double Pore Slim electrode, Hamilton Company, Reno, NV, USA) and A_650_ values were measured. Supernatants were collected (12,000× *g*, 5 min, 4 °C) and used to evaluate the production of GABA with thin-layer chromatography (see Section 2.5.1).

Cultures obtained from MRS and MRS-G cultivation, at both 24 h and 48 h, were further used to qualitatively verify the presence of glutamate decarboxylase (GAD) activity through a colorimetric assay [18]. Briefly, cell suspensions (A_650_ of 3.0) were incubated for 24 h at 37 °C in a solution containing 50 mM glutamic acid and 0.1 mM Tween 80, at pH 4.7; at the end of the incubation, the pH values were measured and supernatants (12,000× *g*, 5 min, 4 °C) were mixed (33:1 ratio) with a pH indicator containing 0.1% (*w*/*v*) methyl red and 0.05% (*w*/*v*) methylene blue in 100% (*v*/*v*) ethanol. The color change of the reaction mixture, from magenta (pH < 5.4) to green (pH > 5.4), was annotated and recognized as potential GAD activity. Two biological replicates were carried out for each strain and treatment.

### 2.3. Effect of Cultivation on GABA Production: A Screening Step

The MRS precultures (16 h, 30 °C) of five selected strains (PB13L, B02, B29, F02, B24) were standardized (A_650_ of 1.0) and used to inoculate (2% *v*/*v*) mWMB (suitable medium for aerobic and respiratory cultivation; [17]) with or without MSG (10 g/L) supplementation. Inoculated mWMB and mWMB-G media were incubated under anaerobic (AN; static growth in screw-cap bottles, AnaeroGen sachets, Oxoid), aerobic (AE; shaken flasks filled on a rotary shaker at 150 rpm) and respiratory (RS; aerobic growth with 2.5 µg/mL hemin and 1.0 µg/mL menaquinone supplementation, rotary shaker at 150 rpm) conditions at 30 °C for 24 h. Samples were aseptically withdrawn at 1 h intervals for the measurement of pH and A_650_ values and for the detection of consumed glutamate and produced GABA by TLC (Section 2.5.1) and RP-HPLC (Section 2.5.2) analyses. A standard curve correlating A_650_ and cell dry weight (CDW, g/L; washed biomass was dried at 105 °C for 24 h) was used to estimate the biomass concentration. Growth curve parameters (i.e., lag phase, maximum specific growth rate) were obtained by modeling the strain growth with the dynamic model of Baranyi and Roberts [19], using DMFit v3.5 for Excel [20]. Two biological replicates were carried out for each strain and treatment.

### 2.4. Production of GABA from Free and Immobilized Cells of Lvb. brevis LB12 Cultivated under Anaerobic and Aerobic Conditions

#### 2.4.1. Production of Free and Immobilized Cells 

*Lvb. brevis* LB12, recognized as the best GABA-producer, was used for further trials. Cells, cultivated under AN and AE conditions (in mWMB supplemented with 10 g/L of MSG, as described in Section 2.3), were washed twice (8000 rpm, 15 min, 4 °C) with phosphate buffer, 20 mM, pH 7 (PB7), standardized to a final biomass of 1 g/L (3.10 × 10^9^ cfu/mL) or 10 g/L (3.10 × 10^10^ cfu/mL) and used for the respective production of free or immobilized cells. For the latter, the standardized suspensions (10 g/L) were mixed (1:9 ratio) with sterile sodium alginate solution (2% *w*/*v*) to obtain a final biomass of 1 g/L, stirred at 150 rpm and added drop-wise to a sterile 200 mM calcium chloride solution. The formed beads were stirred at 150 rpm for 30 min in calcium chloride solution to allow hardening. Beads were collected, washed with NaCl 0.85% (*w*/*v*), and used to evaluate the production of GABA in the buffer system.

#### 2.4.2. Production of GABA in the Buffer System 

Free (1 g/L final biomass) and immobilized (beads weight:buffer volume = 1:1) cells, obtained by anaerobically (AN) or aerobically (AE) growing cultures (Section 2.4.1), were incubated (37 °C, 4 h, 150 rpm) in 50 mM sodium acetate buffer supplemented with 0.1 mM pyridoxal-5′-phosphate (PLP) and 10 g/L of MSG, pH 4.4. At the end of the incubation, free cells (FC) were collected by centrifugation (8000 rpm, 15 min, 4 °C), while immobilized cells (IC) were recovered by separation on a sterile strainer. The number of survivors (detected by plate counting on WMB agar, pH 6.8) for both free and immobilized cells, as well as the pH values and residual cells in the reaction buffer was verified at the end of the incubation. Glutamate consumption and GABA production in buffer supernatants (12,000× *g*, 5 min, 4 °C) were evaluated by TLC (Section 2.5.1) and RP-HPLC (Section 2.5.2) analyses. Two biological replicates were carried out for each condition.

#### 2.4.3. Production of GABA from Resting Cells

Free and immobilized cells, obtained by anaerobically (AN) or aerobically (AE) growing cultures (Section 2.4.1) and used for GABA production in the buffer system (as described in Section 2.4.2), were collected (centrifugation at 8000 rpm, 15 min, 4 °C for FC; separated on a strainer for IC), washed twice, and stored at 4 °C in NaCl 0.85% (*w*/*v*; for FC) or NaCl 0.85% (*w*/*v*) + 10 mM CaCl_2_ (for IC). At intervals of 10 days and up to 30 days of cold storage (for a total of 3 regeneration cycles), the starved free and immobilized cells were reused to evaluate the production of GABA in the buffer system, as described in Section 2.4.2. The pH values, number of survivors, glutamate consumption, and GABA accumulation were measured at the beginning and end of each bioconversion step (4 h of incubation), as described above (Section 2.4.2).

### 2.5. Detection of GABA with Chromatographic Techniques

#### 2.5.1. Thin-Layer Chromatography

Glutamate consumption and GABA accumulation in culture supernatants (Section 2.3) and buffer samples (Section 2.4.2 and Section 2.4.3) were qualitatively evaluated through a TLC assay (silica gel plates, Sigma—Aldrich, St. Louis, MO, USA). The mobile phase was a mixture of *n*-butanol, acetic acid, and deionized water (4:1:1 ratio), supplemented with 0.2% (*w*/*v*) ninhydrin (pre-stained method) for spot detection. Samples were loaded (1.5 µL/spot) on TLC plates, air-dried, and separated in a TLC chamber at room temperature. After the run, the TLC plates were heated at 70 °C (heating plate, 5 min) for spot visualization (red–purple color). Uninoculated culture media and buffer, with or without MSG (10 g/L) or GABA (10 g/L) supplementation, were used as controls. 

Images of TLC plates were acquired (.tiff) by scanning (Brother DCP-J132W Printer Scanner, Brother Industries, Ltd., Nagoya, Japan; resolution 150 dpi, contrast 36, brightness −80), and densitometric parameters of detected spots were analyzed with NIS-Element BR v2.10 software (Nikon, Amstelveen, The Netherlands). Specifically, size (area, mean area, perimeter) and density (mean density, integral density, mean grey) parameters were acquired and use for statistical analysis and correlation with RP-HPLC data.

#### 2.5.2. Reverse Phase—High-Performance Liquid Chromatography

Glutamate consumption and GABA accumulation in culture supernatants (Section 2.3) and buffer samples (Section 2.4.2 and Section 2.4.3) were quantitatively evaluated through an RP-HPLC assay. All samples were subject to the derivatization protocol, according to Zheng et al. [21]. Samples (10 μL) were vacuum-dried (speed vacuum concentrator, PC 3001 VARIO, VACU BRAND) for 2 h at 65 °C, re-suspended in 20 μL of methanol–water–triethylammonium (TEA; ratio 2:2:1, *v*/*v*/*v*) and re-dried under vacuum conditions at 65 °C for 30 min. Dried samples were mixed with 20 μL of methanol–water–TEA–phenyl isothiocyanate solution (PITC; ratio 7:1:1:1, *v*/*v*/*v*/*v*) and vortexed for 5–10 s; PITC-derivatization was performed at 25 °C for 20 min, after which the excess reagent was removed by vacuum-drying at 65 °C for 30 min. Dried samples were dissolved in 12 μL of 60% acetonitrile aqueous solution and 113 μL of 0.05% formic acid aqueous solution. The mixture was centrifuged at 11,000× *g* for 5 min and filtered with a 0.20 μm cellulose acetate (CA) membrane. Uninoculated culture media and buffers supplemented with different concentrations of MSG (range 0–10 g/L) or GABA (range 0–10 g/L) were also derivatized and used to build standard curves of MSG and GABA. 

All samples were analyzed with a Shimadzu HPLC system (LC-20AB equipped with SPD-M20A Prominence Diode Array Detector, Shimadzu Corporation, Kyoto, Japan), and a Macherey–Nagel C18 reverse-phase column (Macherey–Nagel, Düren, Germany, 4.6 mm × 250 mm × 5 μm). The UV detector was set at 254 nm, and the analysis time was 50 min. The HPLC elution system included the mobile phase A, 0.05% formic acid aqueous solution, and the mobile phase B, 70:30 (*v*/*v*) acetonitrile–water. Samples (20 μL) were injected at a flow rate of 1.0 mL/min and separated with the gradient described in Zheng et al. [21]. Chromatogram analysis was carried out with the LabSolutions software version 5.51 (Shimadzu Corporation, Kyoto, Japan), and the peak area, peak height, and retention time were retrieved for statistical analyses and correlation with TLC data. Two biological and technical replicates were carried out for each condition.

### 2.6. Statistical Analysis

Statistical analyses and graphs were obtained using Systat 13.0 for Windows (Systat Software Inc., San Jose, CA, USA) and R 4.1.2 [22].

## 3. Results

### 3.1. Screening for Glutamate Decarboxylase Activity and GABA Production

**Figure 1:**
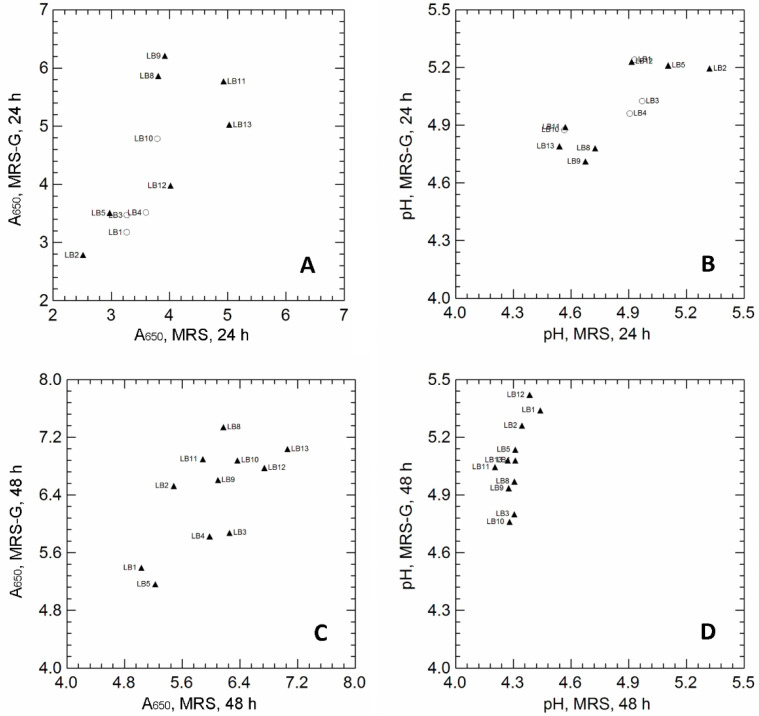
**Panel A** and **B:** Correlation between the A_650_ values of *Levilactobacillus brevis* strains cultivated for 24 h (**A**) and 48 h (**C**) in unsupplemented MRS (x-axis) and MRS supplemented with 10 g/L MSG (MRS-G; y-axis). **Panel B** and **D**: Correlation between pH values measured in unsupplemented MRS (x-axis) and MRS supplemented with 10 g/L MSG (MRS-G; y-axis), inoculated with *Levilactobacillus brevis* strains, after 24 h (**B**) and 48 h (**D**) of incubation. Pearson’s r correlation coefficient: *r* = 0.697 (panel **A**); *r* = 0.758, (panel **B**); *r* = 0.710, (panel **C**); *r* = 0.610, (panel **D**). Symbols refer to the production of GABA, detected by TLC analysis: empty circles, no GABA spot in MRS-G samples at 24 h; filled triangles, GABA spot in MRS-G samples at 24 h; the GABA spot was missing in all unsupplemented MRS samples. Strain code: LB1 is PA11S; LB2 is PB13L; LB3 is TO62; LB4 is A7; LB5 is A4; LB8 is B02; LB9 is B17; LB10 is B25; LB11 is B29; LB12 is F02; LB13 is B24.

Eleven strains of *Lvb. brevis* were cultivated in MRS broth, with or without glutamate (GLU) supplementation (MRS-G, MRS), and tested for the capability to produce GABA. The effect of GLU on the final cell density (A_650_) and pH values is reported in Figure 1.

After 24 h of incubation, GLU supplementation increased the final biomass in only a few strains (LB8, LB9 and LB11; Figure 1A), but did not affect the pH values of the cultivation media (Figure 1B). Regardless of the final cell density, most strains (LB2, LB5, LB8, LB9, LB11, LB12, LB13) were able to produce GABA when cultivated in MRS-G (GABA production is indicated by black triangles in Figure 1A,B; GABA was qualitatively detected on TLC plates; see Supplementary Figures S1 and S2, as examples of TLC images); on the contrary, GABA spots were missing in all MRS supernatants, confirming that GABA was produced exclusively through GLU conversion. Prolonged cultivation (48 h; Figure 1C,D) significantly increased the final cell density, regardless of GLU supplementation. A general increase in pH was observed in the MRS-G supernatants, and all strains were able to produce GABA (TLC spots; black triangles in Figure 1C,D).

The biomass recovered from MRS and MRS-G cultures, at both 24 h and 48 h incubation, was used to verify the presence of possible glutamate decarboxylase (GAD) activity, by using a colorimetric assay based on the color change (from purple at pH < 5.4 to green at pH > 5.4) of a complex pH indicator; the increase in the pH of the reaction mixture was, in fact, qualitatively correlated with glutamate decarboxylation that led to H^+^ consumption and the production of alkaline GABA [1]. pH values were also analytically measured with a pH electrode and used to verify the correlations with color variations (Supplementary Figure S3A,B). After 24 h of incubation, a significant increase in pH was observed in reaction mixture inoculated with LB2, LB8, LB9, LB11, LB12, and LB13 (the strains produced GABA in MRS-G; Figure 1A); however, while the strains LB8, LB9, LB11, and LB13 promoted the color change and the pH increase (purple to green; pH > 5.4) of the reaction mixture, regardless of the growth medium (MRS or MRS-G), LB2 and LB12 led to color and pH variations only when cultivated in MRS-G, suggesting a potential induction of GAD activity in the presence of glutamate. After 48 h of incubation (Supplementary Figure S3B), a significant increase in pH values was observed only in the reaction mixture inoculated with the strains cultivated in MRS-G.

### 3.2. Effect of Cultivation on Biomass Production and GABA Accumulation

*Lvb. brevis* LB8, LB11, and LB13 (high cell density and GABA production after both 24 h and 48 h of incubation, Figure 1A,C; potential GAD activity and the ability to increase the pH values in the buffer system regardless of the growth medium, Supplementary Figure S3) and LB2 and LB12 (high cell density and GABA production after both 24 h and 48 h of incubation, Figure 1A,C; induction of GAD activity by GLU supplementation in the cultivation medium; Supplementary Figure S3) were selected and used to investigate the effect of cultivation (i.e., anaerobiosis, AN; aerobiosis, AE; respiration, RS) on the growth parameters, biomass yield, and GLU/GABA conversion. The growth parameters are shown in Table 1, while data on GLU consumption and GABA production are reported in Table 2 and in Supplementary Figure S4.

The dynamic model of Baranyi and Roberts [19] provided a good fit for all cultivations (R^2^ from 0.940 to 0.999). For some strains (LB2, LB8, LB13), the maximum specific growth rate (µ_max_) was affected by the growth conditions, and the highest values were found in AE and RS cultures (Table 1). The presence of GLU, on the contrary, did not affect the growth rates. Biomass production was affected by both cultivation and GLU supplementation. AE and RS growth significantly (up to 4.2 times) increased the biomass concentration (X_max_) of all strains; the effect of GLU, instead, was strain-specific. Further, the pH values were differently related to the type of cultivation and GLU supplementation.

GLU consumption and GABA production during growth were qualitatively evaluated with TLC assay, and the results are reported in Table 2. At 9 h of incubation, no GABA production was detected (Supplementary Figure S1). GABA spots, in fact, were observed only after 24 h of cultivation (Supplementary Figure S2), suggesting that GABA accumulation was strongly related to a high cell density. Oxygen completely inhibited GABA production in LB2, LB8, and LB11 (spots were clearly evident only under AN conditions), and to a lesser extent, in LB12 and LB13.

RP-HPLC data (Table 2) confirmed that GLU supplementation induced the production of GABA, and for three strains (LB2, LB8, and LB11), aerobic incubation and/or the addition of respiratory cofactors had a detrimental effect. Anaerobically growing cells of LB2, LB8, and LB13 had the highest specific productivity (GABA/biomass), while LB12 and LB13 produced GABA under all growth conditions, although the productivity was very low.

### 3.3. The Production of GABA from Free and Immobilized Cells of Lvb. brevis LB12 Cultivated under Anaerobic and Aerobic Conditions

The strain *Lvb. brevis* LB12, showing the highest ability to produce GABA under aerobic (AE) and respiratory (RS) conditions, was selected and used for further bioconversion trials. The combined effect of oxygen availability (AN vs. AE growth) and cell protection (free vs. alginate-immobilization), as well as the use of resting cells (starved at 4 °C) for GABA production without the cultivation step, was evaluated in the buffer system. As *Lvb. brevis* LB12 had similar growth performances under both AE and RS conditions (Table 1), the AE cells were preferred to the RS ones in order to reduce the cost of biotransformation due to hemin and menaquinone supplementation. 

**Figure 2:**
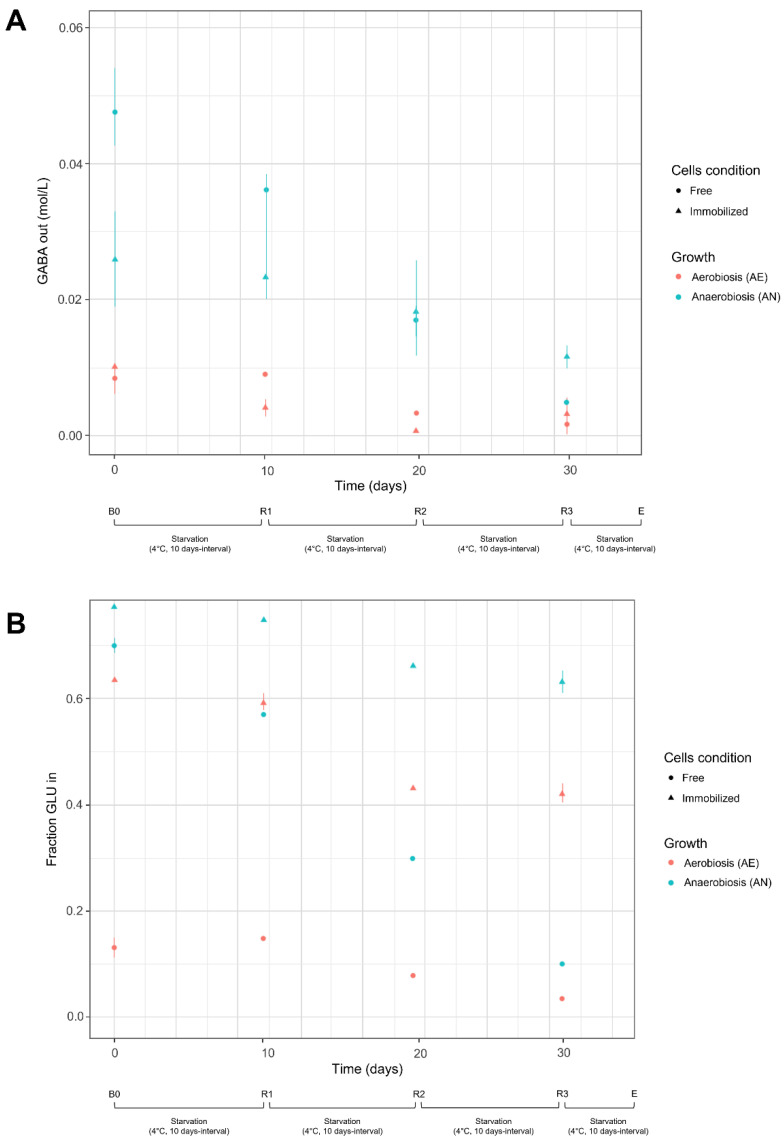
GABA produced (mol/L) and extruded by cells (panel **A**) and fraction of glutamate (expressed as a ratio between initial GLU and GLU transported into the cells) available for bioconversion (panel **B**). Cell condition (free, immobilized) and growth (aerobiosis, anaerobiosis) used for biocatalysis are described in Section 2.4.1 and Section 2.4.2 Bioconversion and recycling steps: B0, first bioconversion step (time: 0 days) carried out with fresh cells; R1, R2, and R3: recycling steps carried-out at 10 days interval (up to 30 days) with resting cells (see Section 2.4.3); E, end of starvation and bioconversion steps.

Fresh AN and AE cells had significant differences in GLU uptake efficiency (Table 3; Figure 2B) in all bioconversion and recycling steps (B0, R1, R2, R3); immobilization in alginate beads, instead, attenuated the effect of cultivation, although the AN cells had the highest GLU transport ability. Consistent with these data, the biotransformation efficiency (GLU to GABA) was higher in AN cells compared to AE ones. However, contrary to GLU uptake efficiency, the differences between AN and AE cultivation were more pronounced in immobilized cells than in free suspensions (Table 3); this behavior could be probably related to a different GABA diffusion outside the alginate beads. In the first step of bioconversion (B0), the AN cells of *Lvb. brevis* LB12 reached 98.76% of GLU transformation, confirming that the strain is an excellent GABA producer. Consistent with data of GABA production, AN free cells had the greatest increase in pH in the reaction buffer (Supplementary Figure S5), while the lower pH variation was observed in AE-immobilized cells.

The AN and AE cultures, in both free and immobilized form, were stored at 4 °C and reused for further biotransformation steps (R1, R2, R3) to evaluate the residual glutamate activity and GABA production ability of resting cells. As expected, the starvation cycles (10 days interval) reduced the efficiency of cells in both GLU uptake and GLU to GABA biotransformation. The ability to transport GLU was mainly impaired in AN free cells and to a lesser extent in immobilized cultures. At the end of the starvation cycles (R3, 30 days), the transport activity of free AN and AE cells was nearly exhausted, and the concentration of GLU available for bioconversion was very low. However, the transformation efficiency was still considerable (71.5% for AN cells; 60.7% for AE cells), as most of the intracellular GLU was converted to GABA (Table 3; Figure 2A; Supplementary Figure S6). On the contrary, the resting immobilized cells maintained a sufficient GLU uptake ability, but the starvation cycles significantly reduced the concentration of produced GABA and then the bioconversion efficiency. However, the low amounts of GABA, detected in the buffer system, could be due to an impaired glutamate decarboxylase activity in resting cells or to a reduced GABA efflux across the alginate coating. Changes in pH during the recycling steps (R1, R2, R3) were consistent with GABA production (Supplementary Figure S5), and the differences in the initial pH values (pH 4.4, buffer) decreased with increasing number of starvation steps.

**Figure 3:**
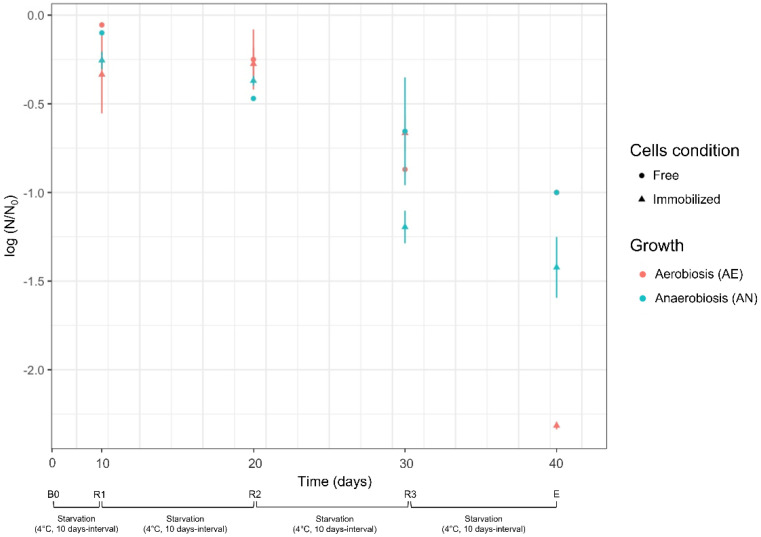
Survival of *Lvb. brevis* LB12 during starvation cycles (4 °C, 10 days intervals). Survival was expressed as the reduction of log N/N_0_, where N_0_ and N are the respective number of cells at the beginning and at the end of each starvation step (t0–t10 for R1; t10–t20 for R2, t20–t30 for R3). Cell condition (free, immobilized) and growth (aerobiosis, anaerobiosis) used for biocatalysis are described in Section 2.4.1 and Section 2.4.2. Bioconversion and recycling steps: B0, first bioconversion step (time: 0 days) carried out with fresh cells; R1, R2, and R3: recycling steps carried out at 10 days intervals (up to 30 days) with resting cells (see Section 2.4.3); E, end of starvation and bioconversion steps.

During the starvation cycles, the survival of free and immobilized cells was also evaluated (Figure 3). *Lvb. brevis* LB12 had high viability (log reduction < the 0.5 cycles) in the first two steps of cold starvation (t0-t10, B0-R1; t10-t20, R1-R2), regardless of cell condition (free or immobilized) and growth (anaerobiosis or aerobiosis). Further starvation cycles (t20-t30, R2-R3; t30-t40; R3-E) led a significant cell reduction, suggesting that prolonged reuse was not beneficial in terms of cell density and bioconversion efficiency. Furthermore, although the cell concentration was reported to be 10^9^ cfu/mL at the beginning of each bioconversion step (at least for free cultures), the starved cells certainly had reduced GAD and GLU/GABA antiport activity. During the starvation cycles, for immobilized cultures, no significant efflux of cells (<9 cfu/mL) across the alginate beads was detected.

**Figure 4:**
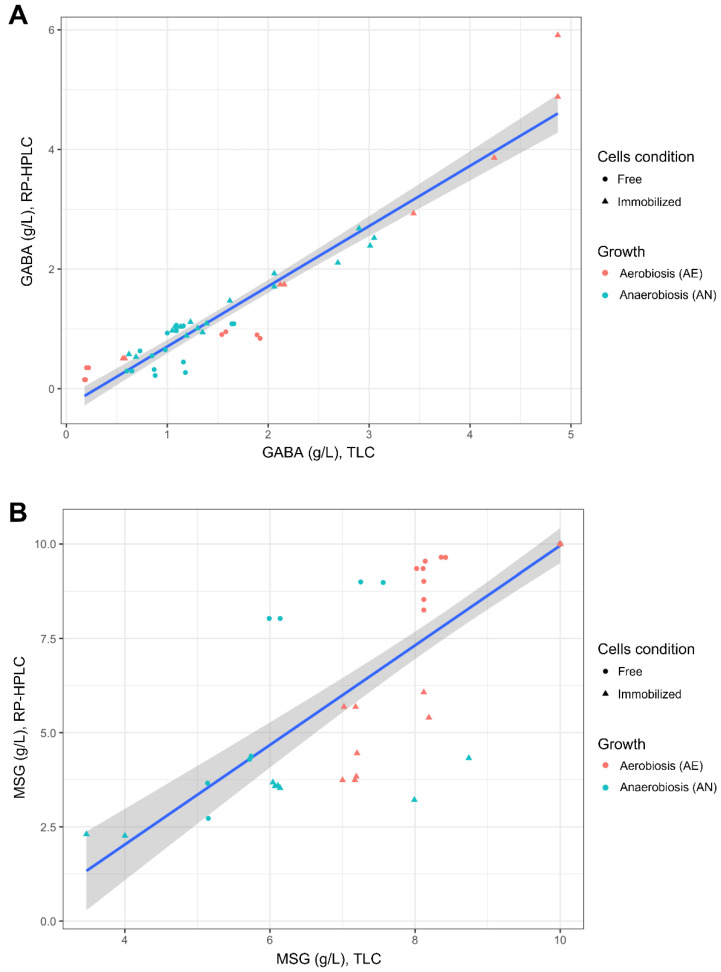
Correlation between the concentrations of produced GABA (g/L; panel **A**) and residual glutamate (g/L; panel **B**) measured in the buffer system by using Thin-layer Chromatography (TLC; Section 2.5.1) and RP-HPLC (Section 2.5.2). The cell condition (free, immobilized) and growth (aerobiosis, anaerobiosis) used for biocatalysis are described in Section 2.4.1 and Section 2.4.2.

In this study, the correlation between TLC and RP-HPLC detection was also evaluated (Figure 4). TLC plates loaded with samples retrieved from the buffer system (Section 2.4.1 and Section 2.4.2) were used for correlation analysis, as the relative images were sharper than those obtained with supernatants from synthetic media (e.g., MRS or mWMB, due to the presence of substrate components). TLC images were analyzed with NIS-Element BR v2.10 software (Supplementary Figure S7), and the integral density (mean density x area) of each spot was used to estimate the concentration (g/L) of consumed GLU or produced GABA. Uninoculated buffer supplemented with different concentrations of GLU or GABA were used as a control. A good correlation was found between TLC and RP-HPLC for GABA detection (Figure 4A), while worse values were observed for GLU estimation (Figure 4A). TLC, however, can be advantageously used as a rapid and semi-quantitative tool for the assessment of GABA production, especially for a high number of samples.

## 4. Discussion

The strains belonging to *Lvb. brevis* are generally recognized as GABA-producing LAB. Members of *Lvb. brevis* are frequently used as starter and/or adjunct cultures in many fermented foods [23,24], and several strains are also exploited as probiotics [25]. The capability to synthetize GABA, therefore, may be beneficial for the production of functional foods and for the formulation of probiotic cultures with psychobiotic effects.

In this study, 11 *Lvb. brevis* were screened for GABA production, and our data confirmed that this ability is widespread among the strains. As expected, GABA accumulation was more evident with increasing cell density, and for some strains, the pre-cultivation in glutamate-supplemented medium (0.69 mM glutamate was added) was needed for the induction of GAD activity, and in some cases, this promoted the growth performances of the strains. However, several studies demonstrated that a high concentration of monosodium glutamate (MSG) may impair cell growth and bioconversion efficiency [9,26]. On the other hand, the bioconversion rate follows the Michaelis–Menten equation at low substrate concentrations (<80 mM; [14,15]).

A rapid and qualitative colorimetric assay was also carried out for the qualitative detection of GAD activity. Strains cultivated in glutamate-supplemented medium led to a greater pH increase and color change in the reaction mixture containing glutamate and a complex pH indicator compared to the strains cultivated in unsupplemented substrate; moreover, the color variations of the pH indicator were consistent with pH values measured analytically, suggesting that the colorimetric assay can be a quick tool for testing a large number of strains. Several authors [18,27] observed a considerable increase in pH in culture medium supplemented with MSG because of glutamate to GABA conversion. The consumption of an intracellular proton H^+^ during the decarboxylation step and the exchange of extracellular glutamate with the more alkaline GABA, in fact, may cause an increase in the extracellular pH. A significant increase in pH was also observed in this study during the bioconversion cycles (Supplementary Figure S5), confirming that the mild alkalization of medium is due to the GAD activity of GAD and GABA production.

The efficiency of glutamate to GABA conversion depends on several factors and to date, the available data are mainly related to the effect of pH, T °C, substrate composition, glutamate, and PLP concentrations on strain growth, the biocatalysis process, and GABA production. To our knowledge, the effect of the atmosphere of incubation (i.e., oxygen availability) on the GABA system has been poorly investigated [11]. Similar to other LAB species [13], *Lvb. brevis* includes several oxygen-tolerant strains, for which aerobic and respiratory (aerobiosis with heme and menaquinone supplementation) cultivation may promote growth performance and stress robustness [28]. In this study, we confirmed that aerobiosis significantly improved the final cell density, favoring the biomass concentration useful for glutamate to GABA bioconversion. The presence of oxygen, however, significantly impaired the glutamate uptake efficiency and GABA accumulation. Consistent with our results, Wu and Shan [11] demonstrated that aerobic growth (shaken flask cultivation) repressed GABA production in *Lvb. brevis* 145, and the medium acidification (low pH generally promoted GAD activity) did not fully restore the bioconversion ability of the strain under aerobic conditions. qPCR analyses, however, revealed the presence of *gad* operon transcripts in aerobic growing cells, suggesting that the GAD system may still function under aerated conditions. Our data also demonstrated a minimal GABA production in aerated cells, suggesting that the GABA system was not completely inhibited by oxygen. Therefore, the aerobic cultivation could be exploited as a first step for the massive production of biomass, and a subsequent shift to anaerobic conditions could be used to restore the GABA system and ensure a high conversion rate.

The stability and reuse of biocatalysts are important factors to optimize metabolite production and decrease the costs of potential industrial applications. In this study, we investigated the effect of alginate immobilization as a cell protection strategy, and we performed a total of four bioconversion cycles (B0 with fresh cells; R1, R2, R3 with resting cells) for GABA accumulation. The reaction cycles were repeated at 10 day intervals, in which the AN and AE free or immobilized cells were maintained under starved conditions. To our knowledge, this is the first report that evaluated the effect of cold storage on GAD activity and GABA production from resting cells. Previous studies, in fact, addressed the repeated bioconversion cycles without cold storage intervals. Huang et al. [29] first demonstrated that the immobilized cells (sodium alginate coating) of *Lvb. brevis* were useful for GABA production (although cell density > 11 g/L may compromise glutamate and GABA diffusion in beads) and may maintain a high bioconversion efficiency (90%) after five cycles of biocatalysis. Successively, Zhang et al. [15] compared the activity of growing and resting cells, demonstrating that the latter had a conversion rate similar to that produced by purified GAD enzyme, and biotransformation could be repeated at least three times with the same yield levels. Shi et al. [14]) also demonstrated advantages in the use of resting cells compared to growing ones, but the authors did not verify their possible reuse for GABA production. More recently, Lyu et al. [30] performed 10 reuse cycles with immobilized cells (gellan gum coating) of an engineered *Lvb. brevis*, demonstrating that entrapped cells had unchanged conversion efficiency up to four decarboxylation cycles. On the contrary, the reusability of free cells was significantly lower.

Our data, in contrast, demonstrated that immobilization did not improve GABA production, while the reuse of starved cells contributed to further GABA accumulation, especial in AN free cells. Therefore, although the use and recycling of resting cultures may be a potential strategy to improve GABA production, overcoming the constraints of strain cultivation and/or enzyme purification, further investigations are needed to reach and maintain high conversion rates after prolonged reuse steps. Additionally, coating agents with a reduced diffusion barrier should be tested in order to ensure efficient glutamate and GABA efflux across beads.

In our study, the consumed glutamate and produced GABA were measured by using both TLC and RP-HPLC methods. TLC is widely used for GABA detection, but to our knowledge, this is the first report that correlates glutamate and GABA concentrations estimated with TLC assay (an accurate image analysis is needed) with data obtained by quantitative RP-HPLC. Furthermore, the use of a pre-stained TLC protocol allowed us to obtain more resolved samples and cleaner images compared to the traditional TLC (staining following the running step). Our results, however, demonstrated that the TLC assay provided more resolved spots for GABA than for GLU, and thus a greater correlation was observed between the GABA content detected with TLC and RP-HPLC. Some smeared spots, in fact, were observed for GLU (the GABA ones were more defined and rounder), and this could have compromised the correct definition of some spot parameters (e.g., mean density, integral density).

However, in general, our data demonstrated that the pre-stained TLC (coupled to a proper image analysis), may be used as a rapid and cost-efficient semi-quantitative tool for the detection of GABA in a large number of samples.

## 5. Conclusions

Our study provides further insights on factors that may affect GABA production in *Lvb. brevis*. The obtained data confirmed that the ability to produce GABA is widespread among the *Lvb. brevis* strains. The strain LB12 is an excellent GABA producer, and the aerobic cultivation may be exploited to improve the final cell density suitable for glutamate to GABA conversion. The recycling and use of resting cells may be exploited to accumulate additional GABA without a cell cultivation step, while immobilization did not improve cell viability and GABA production compared to non-immobilized cultures. Moreover, we demonstrated that pre-stained TLC assay may be a rapid and semi-quantitative assay to evaluate the GABA content in a large number of samples and screening steps. However, given the growing demand for GABA obtained through microbial conversion rather than via chemical synthesis, further investigations are needed to boost and optimize productivity by suitable *Lvb. brevis* strains.

## Figures and Tables

**Table 1:** Growth parameters of *Levilactobacillus brevis* strains cultivated under anaerobic, aerobic, and respiratory conditions, with or without glutamate supplementation.

N	Strain	Growth	MSG	lag	µmax	Xmax	pH
LB2	*Lvb. brevis* PB13L	AN	0	0	0.43 ± 0.03	2.14 ± 0.05	5.90 ± 0.02
			1	-	0.44 ± 0.01	3.13 ± 0.04 *	4.81 ± 0.06 *
		AE	0	-	0.63 ± 0.02 ^†^	7.62 ± 0.04 ^†^	3.94 ± 0.01 ^†^
			1	-	0.63 ± 0.01 ^§^	7.63 ± 0.05 ^§^	4.44 ± 0.00 *^§^
		RS	0	-	0.57 ± 0.02 ^†^	9.06 ± 0.01 ^†‡^	3.92 ± 0.01 ^†^
			1	-	0.56 ± 0.00 ^§^	8.61 ± 0.03 *^§‡^	4.45 ± 0.02 *^§^
LB8	*Lvb. brevis* B02	AN	0	-	0.37 ± 0.01	2.16 ± 0.03	5.10 ± 0.01
			1	-	0.38 ± 0.00	2.33 ± 0.04	4.95 ± 0.04
		AE	0	-	0.58 ± 0.02 ^†^	8.61 ± 0.05 ^†^	4.01 ± 0.03 ^†^
			1	-	0.60 ± 0.01 ^§^	8.30 ± 0.03 ^§^	4.93 ± 0.06 *
		RS	0	-	0.63 ± 0.00 ^†^	9.01 ± 0.02 ^†^	4.02 ± 0.01 ^†^
			1	0.47 ± 0.06 *^§‡^	0.54 ± 0.01 ^§^	9.87 ± 0.05 *^§^	4.49 ± 0.01 *^§‡^
LB11	*Lvb. brevis* B29	AN	0	-	0.56 ± 0.00	3.36 ± 0.04	4.17 ± 0.03
			1	-	0.51 ± 0.02	4.13 ± 0.07 *	4.94 ± 0.04 *
		AE	0	1.47 ± 0.08 *^†‡^	0.61 ± 0.01	8.18 ± 0.04 ^†^	3.87 ± 0.02 ^†^
			1	-	0.62 ± 0.02	8.20 ± 0.05 ^§^	4.37 ± 0.00 *^§^
		RS	0	-	0.56 ± 0.02	8.39 ± 0.01 ^†^	3.84 ± 0.01 ^†^
			1	0.97 ± 0.07 *^§‡^	0.61 ± 0.00 *	10.01 ± 0.05 *^§‡^	4.39 ± 0.01 *^§^
LB12	*Lvb. brevis* F02	AN	0	-	0.43 ± 0.00	2.94 ± 0.01	5.75 ± 0.00
			1	-	0.46 ± 0.00 *	2.66 ± 0.05	5.85 ± 0.02
		AE	0	-	0.46 ± 0.02	6.47 ± 0.06 ^†^	4.00 ± 0.00 ^†^
			1	-	0.46 ± 0.05	6.29 ± 0.04 ^§^	4.56 ± 0.01 *^§^
		RS	0	-	0.42 ± 0.00	6.59 ± 0.04 ^†^	4.01 ± 0.02 ^†^
			1	-	0.44 ± 0.01	6.15 ± 0.02 *^§^	4.54 ± 0.00 *^§^
LB13	*Lvb. brevis* B24	AN	0	-	0.41 ± 0.00	3.04 ± 0.02	4.49 ± 0.00
			1	-	0.36 ± 0.01 *	3.28 ± 0.09	5.46 ± 0.03 *
		AE	0	0.44 ± 0.06 *^†^	0.61 ± 0.01 ^†^	7.57 ± 0.09 ^†^	4.07 ± 0.00 ^†^
			1	-	0.55 ± 0.00 ^§^	7.81 ± 0.05 ^§^	4.55 ± 0.01 *^§^
		RS	0	1.20 ± 0.03 *^†‡^	0.58 ± 0.00 ^†^	9.04 ± 0.11 ^†‡^	4.09 ± 0.02 ^†^
			1	0.75 ± 0.06 *^§‡^	0.53 ± 0.02 ^§^	8.80 ± 0.04 ^§‡^	4.51 ± 0.01 *^§^

N: code of *Levilactobacillus brevis* strains. Growth: AN, anaerobiosis; AE, aerobiosis; RS, respiration. MSG: 0, absence; 1, supplementation of mWMB with monosodium glutamate (MSG, 10 g/L). lag: lag phase (h), estimated value ± standard error. µ_max_: maximum specific growth rate (h^−1^), estimate value ± standard error. The R^2^ of the fit ranged from 0.940 to 0.999. X_max_: maximum biomass concentration (g/L) measured after 24 h of incubation, estimated value ± standard error. pH: pH measured after 24 h of incubation, estimated value ± standard error. * Significant differences (*p* < 0.005, Tukey’s HSD multiple comparisons) between supplemented and unsupplemented mWMB under the same growth condition;^†^significant differences (*p* < 0.005) between AN and AE or RS growth in unsupplemented mWMB; ^§^, significant differences (*p* < 0.005) between AN and AE or RS growth in supplemented mWMB; ^‡^ significant differences (*p* < 0.005) between AE and RS conditions within the same medium.

**Table 2:** The production of γ-aminobutyric acid in *Levilactobacillus brevis* strains cultivated under anaerobic, aerobic, and respiratory conditions, with or without glutamate supplementation.

N	Strain	Growth	MSG	GLU Spot	GABA Spot	GLU HPLC	GABA HPLC	GABA/X
LB2	*Lvb. brevis* PB13L	AN	0	0	0	-	-	
			1	0	1	0.37 ± 0.07	8.21 ± 0.35	2.63 ± 0.10
		AE	0	0	0	-	-	
			1	1	0	10.03 ± 0.95 ^†^	0 ^†^	-
		RS	0	0	0	-	-	
			1	1	0	10.01 ± 0.69 ^†^	0 ^†^	-
LB8	*Lvb. brevis* B02	AN	0	0	0	-	-	-
			1	0	1	0	6.19 ± 0.93	2.67 ± 0.44
		AE	0	0	0	-	-	-
			1	1	1	7.86 ± 0.41 ^†^	1.28 ± 0.03 **^†‡^**	0.15 ± 0.00 ^†^
		RS	0	0	0	-	-	-
			1	1	0	9.96 ± 2.53 ^†^	0 ^†^	-
LB11	*Lvb. brevis* B29	AN	0	0	0	-	-	-
			1	0	1	0	8.36 ± 0.41	2.03 ± 0.12
		AE	0	0	0	-	-	-
			1	1	0	10.00 ± 1.28 ^†^	0 ^†^	-
		RS	0	0	0	-	-	-
			1	1	0	9.86 ± 0.34 ^†^	0 ^†^	-
LB12	*Lvb. brevis* F02	AN	0	0	0	-	-	
			1	1	1	3.36 ± 0.72	5.71 ± 0.84	2.15 ± 0.35
		AE	0	0	0	-	-	
			1	1	1	7.62 ± 0.14 ^†^	2.53 ± 0.24 ^†^	0.40 ± 0.04 ^†^
		RS	0	0	0	-	-	
			1	1	1	7.85 ± 0.20 ^†^	2.29 ± 0.36 ^†^	0.37 ± 0.06 ^†^
LB13	*Lvb. brevis* B24	AN	0	0	0	-	-	
			1	0	1	0	8.61 ± 1.83	2.62 ± 0.53
		AE	0	0	0	-	-	
			1	1	1	6.78 ± 1.22 ^†^	2.70 ± 0.51 ^†^	0.35 ± 0.06 ^†^
		RS	0	0	0	-	-	
			1	1	1	7.97 ± 0.96 ^†^	1.96 ± 0.16 ^†^	0.22 ± 0.02 ^†^

N: code of *Levilactobacillus brevis* strains. Growth: AN, anaerobiosis; AE, aerobiosis; RS, respiration. MSG: 0, absence; 1, supplementation of mWMB with monosodium glutamate (MSG, 10 g/L). GLU spot and GABA spots: absence (0) or presence (1) of glutamate and γ-aminobutyric acid spots on TLC chromatogram (from samples at 24 h of incubation). GLU-HPLC and GABA-HPLC: concentrations (g/L) of GLU and GABA measured with RP-HPLC in the supernatants at 24 h of incubation (HPLC analysis was carried out only in MSG-supplemented medium). GABA/X: GABA concentration (g/L) standardized on biomass production (g/L). ^†^ Significant differences (*p* < 0.005) between AN and AE or RS growth in glutamate supplemented mWMB; ^‡^ significant differences (*p* < 0.005) between AE and RS conditions within the same medium.

**Table 3:** Parameters of glutamate to GABA conversion in free and immobilized cells cultivated undern anaerobic and aerobic conditions.

Cond	Growth	Cycle ^a^	Days ^b^	GLU in (mM) ^c^	GABA out (mM) ^d^	GLU Uptake Efficiency (%) ^e^	Bioconversion Efficiency (%) ^f^
Free cells	Anaerobiosis	B0	0	48.51 ± 1.32	47.91 ± 0.82	71.38 ± 1.94	98.76 ± 0.99
		R1	10	38.50 ± 0.38	36.80 ± 0.89	56.65 ± 0.57	95.57 ± 1.36
		R2	20	20.19 ± 0.00	17.07 ± 0.27	29.70 ± 0.00	84.55 ± 1.36
		R3	30	6.85 ± 0.07	4.90 ± 0.00	10.08 ± 0.11	71.52 ± 0.75
	Aerobiosis	B0	0	9.55 ± 0.58 *	8.44 ± 0.41 *	14.05 ± 0.85 *	88.38 ± 1.03 *
		R1	10	9.99 ± 0.14 *	8.80 ± 0.03 *	14.70 ± 0.21 *	88.09 ± 0.93 *
		R2	20	4.42 ± 0.00 *	3.39 ± 0.00 *	6.50 ± 0.00 *	76.83 ± 0.00 *
		R3	30	2.40 ± 0.02 *	1.45 ± 0.00 *	3.53 ± 0.04 *	60.72 ± 0.61 *
Immobilized cells	Anaerobiosis	B0	0	48.70 ± 0.29	25.14 ± 1.20 ^§^	71.65 ± 0.42	51.63 ± 2.77 ^§^
		R1	10	46.01 ± 0.48 ^§^	23.81 ± 0.89 ^§^	67.70 ± 0.71 ^§^	51.75 ± 2.48 ^§^
		R2	20	43.75 ± 0.31 ^§^	17.60 ± 1.51	64.38 ± 0.46 ^§^	40.22 ± 3.16 ^§^
		R3	30	42.34 ± 5.33 ^§^	5.36 ± 0.31	62.30 ± 7.85 ^§^	12.71 ± 0.87 ^§^
	Aerobiosis	B0	0	42.55 ± 0.00 *^§^	10.52 ± 0.00 *^§^	62.60 ± 0.00 *^§^	24.73 ± 0.00 *^§^
		R1	10	39.79 ± 2.98 ^§^	4.44 ± 0.17 *^§^	58.55 ± 4.38 ^§^	11.16 ± 0.41 *^§^
		R2	20	29.33 ± 0.00 *^§^	2.86 ± 0.00 *^§^	43.15 ± 0.00 *^§^	9.75 ± 0.00 *^§^
		R3	30	28.99 ± 3.22 *^§^	2.62 ± 0.69 *^§^	42.65 ± 4.74 *^§^	9.22 ± 3.39 *^§^

^a^ Bioconversion and recycling steps: B0, first bioconversion step (time 0 days); R1, R2, and R3: recycling steps carried out at 10- day intervals (up to 30 days) with resting cells (see Section 2.4.3). ^b^ time (days) of bioconversion steps carried-out with fresh (B0) and resting cells (R1, R2, R3). ^c^ Concentration of glutamate (GLU) taken up by cells and available for bioconversion (calculated as initial GLU—residual GLU in the buffer system). ^d^ Concentration of GABA produced and extruded by cells (GABA detected in the buffer system). ^e^ Percentage ratio between initial GLU and GLU transported into the cell. ^f^ Percentage ratio of GLU (in) converted in GABA (out). * Significant differences (*p* < 0.005, Tukey’s HSD multiple comparisons) between anaerobic and aerobic cells under the same cell condition (free or immobilized) and bioconversion cycles (B0, or R1, or R2, or R3); ^§^ significant differences (*p* < 0.005) between free and immobilized cells under the same growth condition (anaerobiosis or aerobiosis) and bioconversion cycles (B0, or R1, or R2, or R3).

## Data Availability

Not applicable.

## Supplementary Materials

The following supporting information can be downloaded at https://www.mdpi.com/article/10.3390/microorganisms10112184/s1, Figures S1 and S2: Example of Thin-layer Chromatography (TLC) plate; Figure S3: Correlation between pH values measured in the buffer system inoculated with *Levilactobacillus brevis* strains grown for 24 h (panel **A**; *r* = 0.728) or 48 h (panel **B**; *r* = 0.285) in unsupplemented MRS or MRS supplemented with 10 g/L MSG (MRS-G); Figure S4: Consumed GLU and total and specific GABA production in five selected strains of *Lvb. brevis* cultivated under AN, AE, and RS conditions; Figure S5: Changes in pH during the GLU to GABA bioconversion cycles in the buffer system; Figure S6: Correlation between GABA produced (mol/L) and extruded by cells and glutamate taken up by cells (mol/L) and available for GLU/GABA bioconversion; Figure S7: Example of the image analysis of a TLC plate (experiment in the buffer system; Section 2.4.2 and Section 2.4.3.) by using NIS-Element BR v2.10 software (Nikon, The Netherlands).
