# Supplementary material for: The Production of γ-Aminobutyric Acid from Free and Immobilized Cells of Levilactobacillus brevis Cultivated in Anaerobic and Aerobic Conditions
Source: Microorganisms. 2022 Nov 3;10(11):2184. doi: 10.3390/microorganisms10112184 (PMC9699244; doi:10.3390/microorganisms10112184)
Supplement: Supplementary file 1 [file microorganisms-10-02184-s001.zip › microorganisms-1984267-supplementary.pdf]

Title

Production of γ-aminobutyric acid from free and immobilized cells of *Levilactobacillus brevis* cultivated in anaerobic and aerobic conditions

Authors

Teresa Zotta [1,*], Immacolata Faraone [2,3], Marilisa Giavalisco [1], Eugenio Parente [1], Ludovica Lela [2], Livia Vanessa Storti [1], Annamaria Ricciardi [1]

Affiliations

[1] Scuola di Scienze Agrarie, Alimentari, Forestali ed Ambientali (SAFE), Università degli Studi della Basilicata, 85100 Potenza, Italy

[2] Dipartimento di Scienze (DIS), Università degli Studi della Basilicata, 85100 Potenza, Italy

[3] Spinoff BioActiPlant s.r.l., Viale Dell'ateneo Lucano 10, 85100 Potenza, Italy

* Correspondence: teresa.zotta@unibas.it; Tel +39-0971-205563.

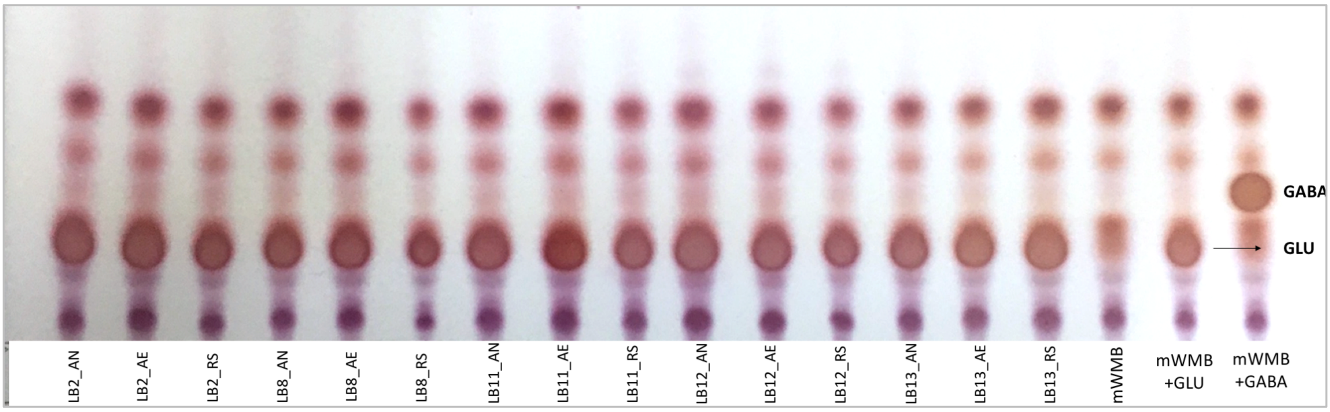

Supplementary Figure S1. Example of Thin Layer Chromatography (TLC) plate. Samples refer to the glutamate consumption and GABA production after 9 h of incubation in the growth condition described in section 2.3 and Table 1. mWMB+GLU and mWMB+GABA (used as controls) are, respectively, the mWMB media supplemented with 10 g/L of glutamate or GABA. Strain code: LB2 is PB13L; LB8 is B02; LB11 is B29; LB12 is F02; LB13 is B24.

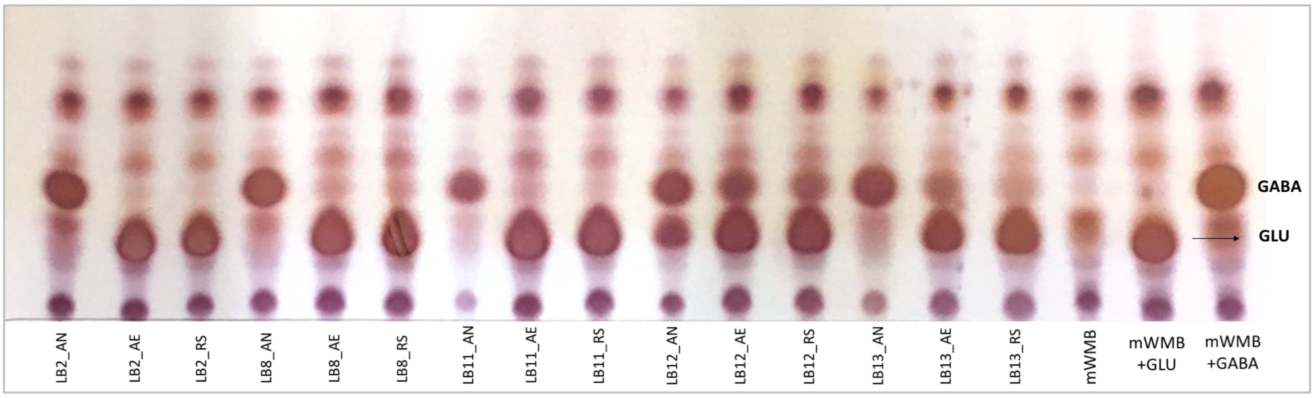

Supplementary Figure S2. Example of Thin Layer Chromatography (TLC) plate. Samples refer to the glutamate consumption and GABA production after 24 h of incubation in the growth condition described in section 2.3 and Table 1. mWMB+GLU and mWMB+GABA (used as controls) are, respectively, the mWMB media supplemented with 10 g/L of glutamate or GABA. Strain code: LB2 is PB13L; LB8 is B02; LB11 is B29; LB12 is F02; LB13 is B24.

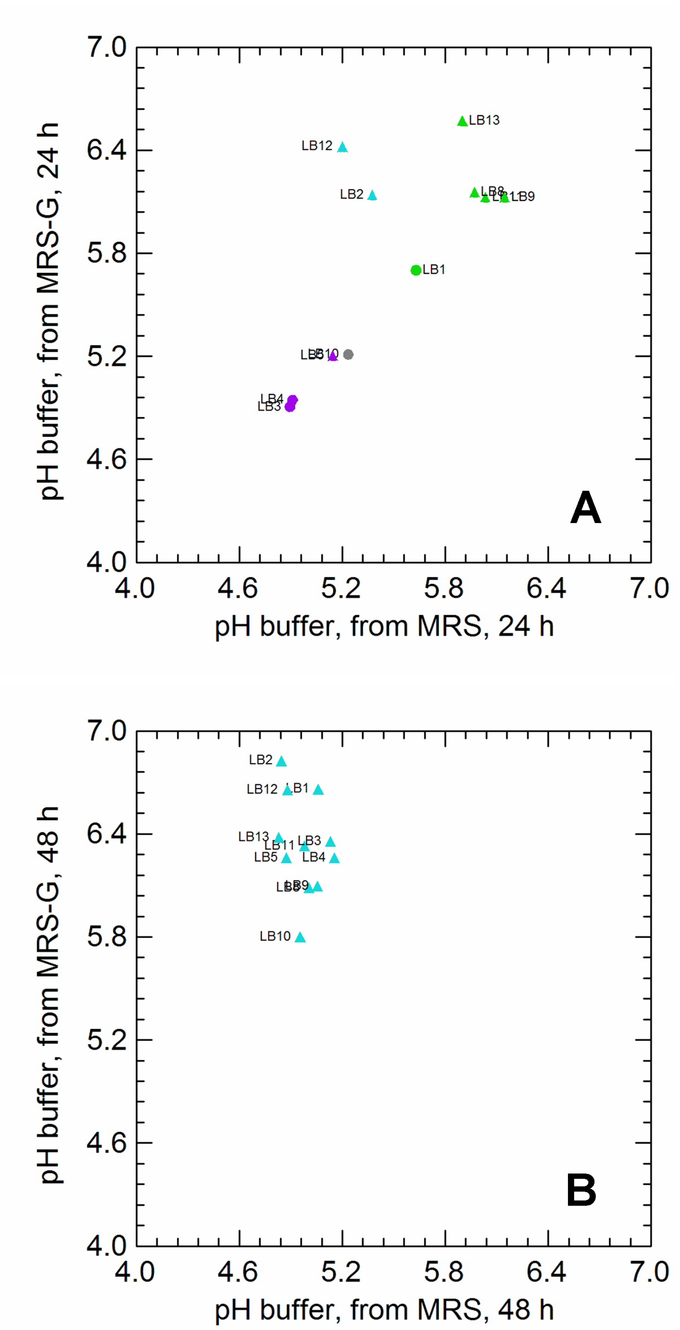

Supplementary Figure S3. Correlation between pH values measured in buffer system inoculated with *Levilactobacillus brevis* strains grown for 24 h (panel **A**; *r* = 0.728) or 48 h (panel **B**; *r* = 0.285) in unsupplemented MRS or MRS supplemented with 10 g/L MSG (MRS-G). Colour of symbols indicates the range of colour and pH values of the reaction buffer inoculated with cells grown in MRS or MRS-G: purple, no colour change (pH < 5.0) in presence of both MRS and MRS-G cultures; grey, colour change from purple to grey (5.0 < pH <5.4) in presence of both MRS and MRS-G cultures; green, colour change from grey to green (pH > 5.4) in presence of both MRS and MRS-G cultures; light blue, colour change from grey to green (pH > 5.4) only in presence of MRS-G cultures. Symbols are referred to the GABA-spots detected each strain after 24 h and 48 h of incubation, as described in **Figures 1A and 1C**, respectively. Strain code: LB1 is PA11S; LB2 is PB13L; LB3 is TO62; LB4 is A7; LB5 is A4; LB8 is B02; LB9 is B17; LB10 is B25; LB11 is B29; LB12 is F02; LB13 is B24.

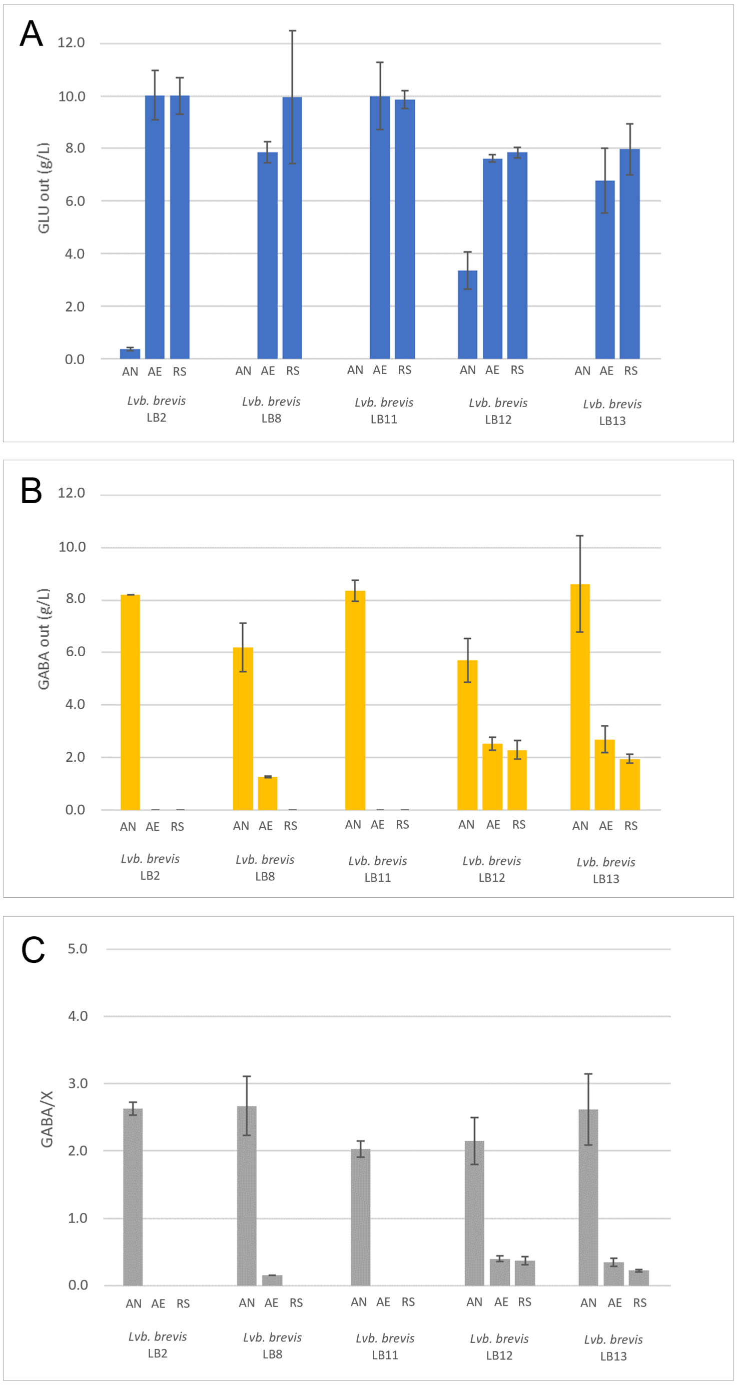

Supplementary Figure S4. Panel **A**: glutamate (GLU out, g/L) measured with RP-HPLC in culture supernatants afetr 24 h of incubation of 5 selected *Lvb. brevis* strains (LB2, LB8, LB11, LB12, LB13); panel **B**: GABA produced (g/L) and extruded by cells and measured with RP-HPLC in culture supernatants after 24 h of incubation of 5 selected *Lvb. brevis* strains (LB2, LB8, LB11, LB12, LB13); panel **C**: specific GABA production (GABA/X, g/L), calculated as ratio between GABA concentration and biomass production (g/L).

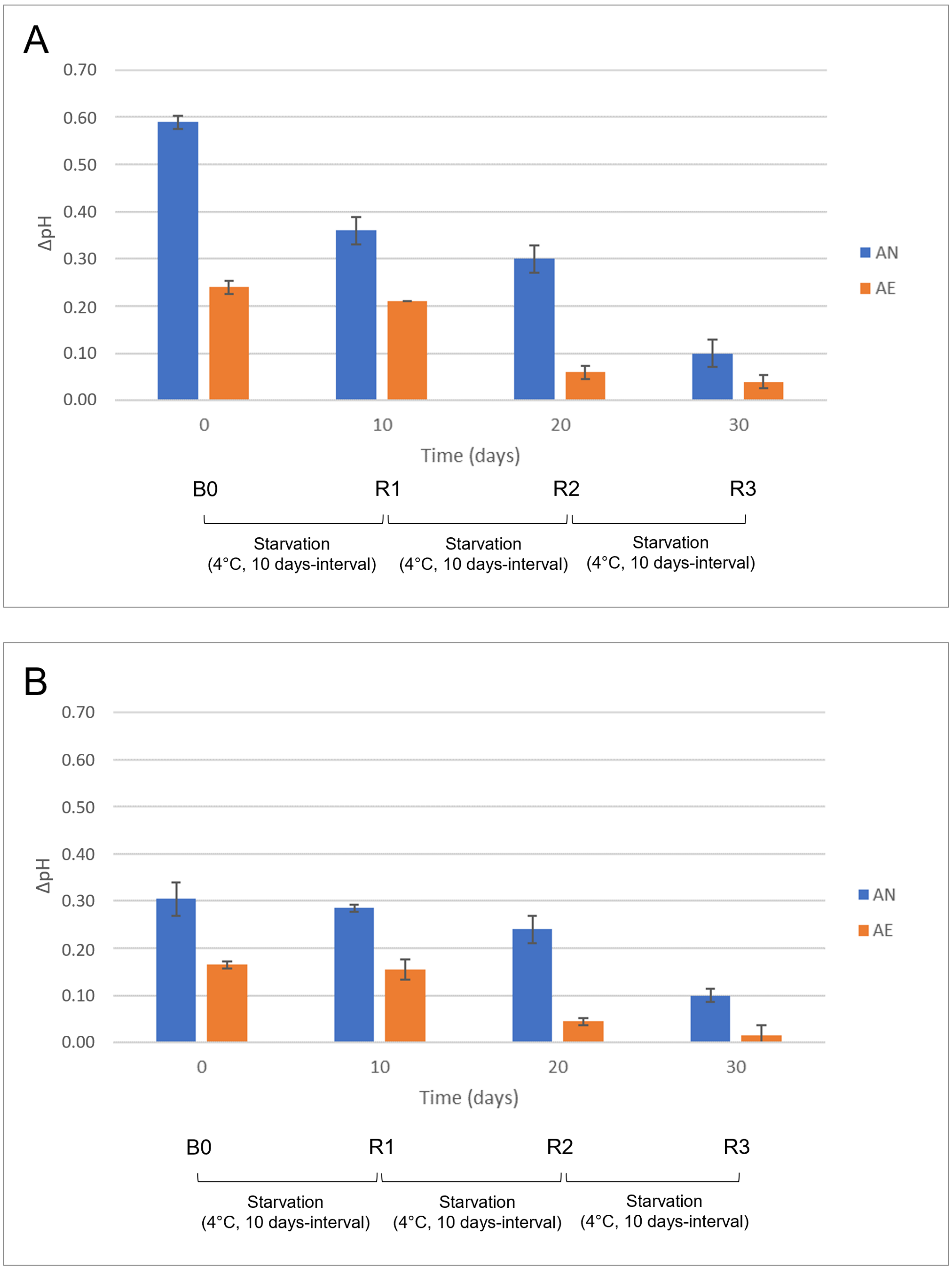

Supplementary Figure S5. Increase of pH measured in the reaction buffer after 4 h of incubation at 37°C (sections 2.4.1) for free (panel **A**) and immobilized (panel **B**) cells. Cell condition (free, immobilized) and growth (aerobiosis, anaerobiosis) used for biocatalysis are described in sections 2.4.1 and 2.4.2. Bioconversion and recycling steps: B0, first bioconversion step (time 0 days) carried-out with fresh cells; R1, R2 and R3: recycling steps carried-out at 10-days interval (up to 30 days) with resting cells (see section 2.4.3); E, end of starvation and bioconversion steps.

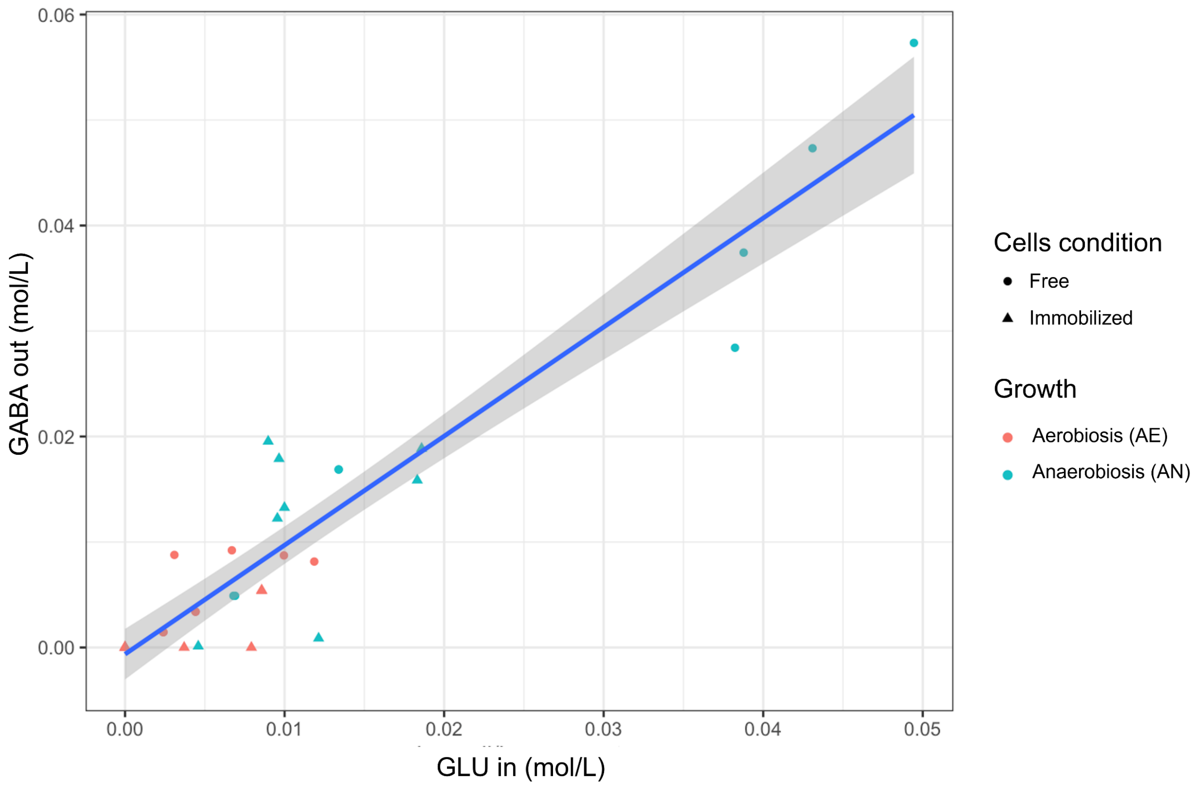

Supplementary Figure S6. Correlation between GABA produced (mol/L) and extruded by cells and glutamate uptaken by cells (mol/L) and available for GLU/GABA bioconversion. Cell condition (free, immobilized) and growth (aerobiosis, anaerobiosis) used for biocatalysis are described in sections 2.4.1 and 2.4.2.

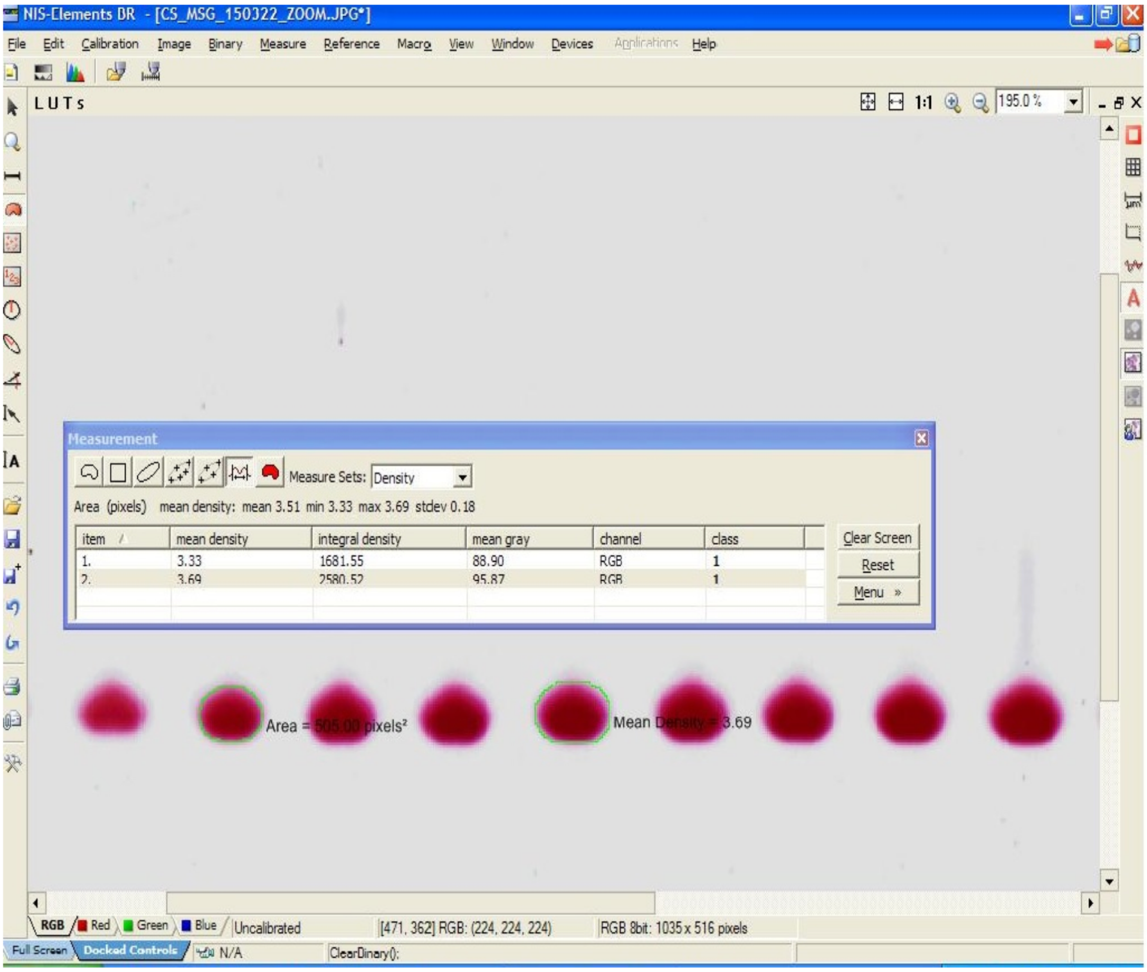

Supplementary Figure S7. Example of the image analysis of a TLC plate (experiment in buffer system; sections 2.4.2 and 2.4.3.) by using the NIS-Element BR v2.10 software (Nikon, The Netherlands). Data from image analysis were correlated with RP-HPLC data.
